# Examining the Trends in Online Health Information–Seeking Behavior About Chronic Obstructive Pulmonary Disease in Singapore: Analysis of Data From Google Trends and the Global Burden of Disease Study

**DOI:** 10.2196/19307

**Published:** 2021-10-18

**Authors:** Yang Fang, Thomas A Shepherd, Helen E Smith

**Affiliations:** 1 Lee Kong Chian School of Medicine Nanyang Technological University Singapore Singapore Singapore; 2 School of Medicine Keele University Staffordshire United Kingdom

**Keywords:** online health information seeking, infodemiology, Google Trends, Global Burden of Disease study, chronic obstructive pulmonary disease, respiratory health

## Abstract

**Background:**

Chronic obstructive pulmonary disease (COPD) is the third leading cause of death globally, and timely health care seeking is imperative for its prevention, early detection, and management. While online health information–seeking behavior (OHISB) is increasingly popular due to widespread internet connectivity, little is known about how OHISB for COPD has changed in comparison with the COPD disease burden, particularly at a country-specific level.

**Objective:**

This study aimed to examine the trends in OHISB for COPD and how that compared with the estimates of COPD disease burden in Singapore, a highly wired country with a steadily increasing COPD disease burden.

**Methods:**

To examine the trends in OHISB for COPD, we performed Prais-Winsten regression analyses on monthly search volume data for COPD from January 2004 to June 2020 downloaded from Google Trends. We then conducted cross-correlational analyses to examine the relationship between annualized search volume on COPD topics and estimates of COPD morbidity and mortality reported in the Global Burden of Disease study from 2004 to 2017.

**Results:**

From 2004 to 2020, the trend in COPD search volume was curvilinear (β=1.69, *t*_194_=6.64, *P<*.001), with a slope change around the end of 2006. There was a negative linear trend (β=–0.53, *t*_33_=–3.57, *P*=.001) from 2004 to 2006 and a positive linear trend (β=0.51, *t*_159_=7.43, *P*<.001) from 2007 to 2020. Cross-correlation analyses revealed positive associations between COPD search volume and COPD disease burden indicators: positive correlations between search volume and prevalence, incidence, years living with disability (YLD) at lag 0, and positive correlations between search volume and prevalence, YLD at lag 1.

**Conclusions:**

Google search volume on COPD increased from 2007 to 2020; this trend correlated with the upward trajectory of several COPD morbidity estimates, suggesting increasing engagement in OHISB for COPD in Singapore. These findings underscore the importance of making high-quality, web-based information accessible to the public, particularly COPD patients and their carers.

## Introduction

Chronic obstructive pulmonary disease (COPD) is the third leading cause of death globally [1] and affected approximately 251 million of the world’s population in 2016 [2]. In Singapore, it is the tenth leading cause of death [3] and affects approximately 5.9% of the general population [4] and 26% of the population aged 55 years and above [5]. The number of deaths caused by COPD in the South East Asia region is projected to increase from 1.04 million in 2016 to 1.43 million in 2030 [6]. Despite its significance, COPD still has poor awareness and understanding by both the public and health practitioners [7]. Therefore, understanding whether and how health information on COPD is accessed in proportion to the size of the disease burden is critical.

Barriers to health care seeking in COPD often lead to fewer prompt diagnoses and poorer disease management among those already diagnosed. For instance, misdiagnoses in primary care settings have been attributed to underuse of and lack of expertise with spirometry and COPD diagnostic guidelines [8]. Furthermore, patients’ knowledge of COPD may be suboptimal because of the complexity of its name, not only in English but also in other languages [9]. Arguably, these barriers can be at least partially attributed to inadequate knowledge of COPD, which can be improved by making high-quality health information more accessible. According to the Health Information National Trends Survey in 2012, the internet was the first source of health information for 70% of adult US internet users [10], suggesting its increasing influence as a source of general and potentially, disease-specific health information.

Disease-specific online health information–seeking behavior (OHISB) among patients with COPD has been studied in several US-based surveys. A postal survey of 1077 patients with COPD in 2007-2008 found that 65% had internet access and 25% of this group used the internet to seek information on COPD at least once weekly. Their frequency of seeking COPD information online was associated with experiencing exacerbations or dissatisfaction with health service providers and treatments [11]. A web-based survey of 445 patients with COPD in 2016 found that physicians were the primary source of COPD information followed by the internet. The patients’ online health information needs were primarily related to symptom control and COPD treatments; also, over 60% of the patients had discussed COPD information on the internet with their health care providers [12]. Another web-based survey of 176 COPD patients found that eHealth literacy, defined as the capacity to seek, locate, understand, evaluate, and apply health information from the internet, was higher in patients with more severe COPD [13]. These findings suggest that, at least in the United States, OHISB is common among COPD patients with internet access, and more frequent OHISB appears to be associated with more unmet needs in disease management.

A frequent method for examining OHISB is Google search data [14]. It is freely downloadable from Google Trends, a publicly accessible portal hosting data on aggregate search activity on the Google search engine delineated by time periods and regions. Since Google is the most popular search engine worldwide, its aggregate data are used as a surrogate indicator for OHISB at the population level.

In 2019, Boehm and colleagues [15] published a study using worldwide Google Trends data that found no change in search volume for COPD in the 15 years from 2004 to 2018. Yet, the number of deaths due to COPD was estimated to have increased by 11.7% from 1990 to 2015, despite a decrease of 41.9% in the age-standardized death rate in the general population [16]. This contrasts with other leading causes of death, such as diabetes and stroke, which have evidenced increased search volumes over the same period. The clear divergence between the rising prevalence of COPD and stagnant OHISB pattern for COPD is a cause for concern, but there are limitations in the study’s methods. The analyses by Boehm et al [15] were performed on worldwide COPD search data without accounting for variation in COPD disease burden between countries and other country-level characteristics such as internet coverage; both factors are essential considerations for developing and implementing country-specific policy changes.

Our study improves upon the work by Boehm et al [15] by situating the enquiry within a single country, Singapore, and comparing Google Trends data with COPD disease burden estimates. Singapore is an opportune context for this research because of high internet penetration, popularity of Google search, and high COPD prevalence [4,5]. Furthermore, Singapore has a rapidly aging population with an increasing burden of chronic diseases. With this in mind, this study was designed to address 2 research questions. First, we aimed to examine the trend in OHISB for COPD (indicated by monthly Google search data) in Singapore from 2004 to 2020. Second, we aimed to compare online search volume with disease burden estimates to assess the extent to which OHISB for COPD reflects the disease burden reported in the Global Burden of Disease study (GBD 2017) over time. To our knowledge, this is the first study that examined trends in OHISB in conjunction with disease burden indicators in COPD.

## Methods

### Search Volume Data From Google Trends

Search query data relating to COPD were obtained from Google Trends, an online portal that displays search queries made worldwide on the Google search engine since 2004. The tool aggregates monthly volumes of search queries, delineated by time period and region, into a metric known as relative search interest (RSI). RSI is computed as a function of a search query’s own highest query share. Its values range from 0 to 100, where 100 represents peak popularity. Google Trends excludes duplicate searches made by the same person over a short period of time. Search queries on Google Trends can be defined either as a search term (the exact search query, accounting for plural and singular forms and spelling mistakes) or a topic (groups of terms that share the same concept in any language).

Following the approach in Boehm et al [15], all search queries were defined as a topic and downloaded as monthly search data. In total, there were 198 monthly data points. When annual data were required for comparison with GBD data, the annual averages were computed from monthly averages.

### Disease Burden Data From GBD 2017

The following data relevant to COPD were obtained from GBD 2017: prevalence, incidence, disability-adjusted life years (DALY; the sum of years lived with disability [YLD] and the years of life lost [YLL]), YLD, YLL, number of deaths (mortality). GBD studies are conducted annually by The Institute for Health Metrics and Evaluation, and reports are typically issued every 2 years. These data are accessible online [17]. Currently, the data come from 195 countries and include 354 diseases and injuries. The data sources are diverse, including published literature, hospital and clinical data, surveillance and survey data, and inpatient and outpatient medical records. A detailed description of the methodology of the GBD 2017 can be found in the article by the GBD 2017 Disease and Injury Incidence and Prevalence Collaborators [2]. The most recent and publicly available GBD data were for 2017, and so the time period used for our study spanned from 2004, the first year for which Google Trends data were available, to 2017.

### Statistical Analyses

As noted in previous works [18], performing Google Trend queries with similar parameters at different times can produce somewhat different data. To mitigate this problem, we performed the same query over 7 consecutive days, from June 22, 2020 to June 28, 2020. To assess the reliability of the data, two-way random model intraclass correlation coefficients (ICCs) were computed. The 7 time series data were averaged to produce a single time series for further analyses. Due to the nature of autocorrelated residuals in time series data, the Prais-Winsten estimation method was used to examine the trend in search volume. To compare search volume with disease burden estimates, a cross-correlation function was used to examine the correlation between the 2 annual time series data. The annual time series for search volume was computed by averaging the monthly search volume over the 12 months of each year.

## Results

### Reliability

The reliability of search volume data was moderate for the single time series data (ICC=0.55) and strong for the averaged time series data (ICC=0.90). The subsequent analyses were performed on the averaged time series data.

### Trends in Monthly Search Volume for COPD From January 2004 to June 2020

**Figure 1:**
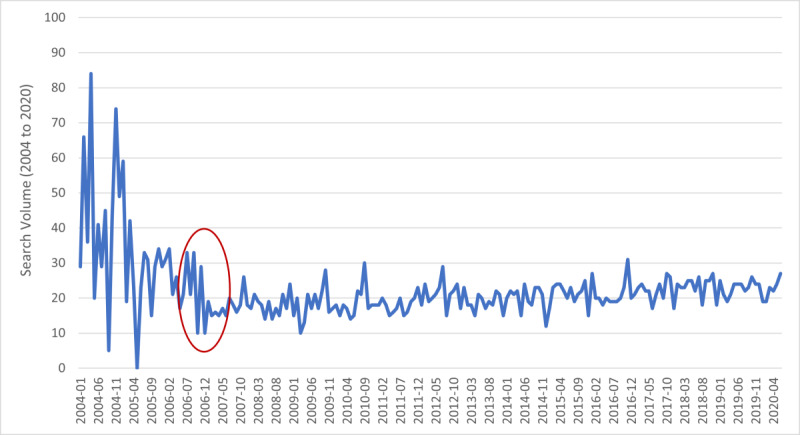
Relative search volume for chronic obstructive pulmonary disease (COPD) from 2004 to 2020. The red circle indicates an apparent slope change in search volume.

The trend in monthly search volume for the COPD topic, shown in Figure 1, was examined using Prais-Winsten regression.

The Durbin Watson statistic [19] was 2.05. The autocorrelation coefficient was 0.09 (SE 0.72). For the overall model, *R*^2^ was 0.22 (SE 8.16). Time demonstrated a quadratic effect on search volume (β=1.69, *t*_194_=6.64, *P*<.001), as shown in Table 1. A quadratic effect suggests that there was 1 slope change over the entire period.

**Table 1:** Prais-Winsten regression examining the effect of time on monthly search volume for chronic obstructive pulmonary disease (COPD; 2004-2020).

Predictors	β^a^	*t* (*df*=194)	*P* value
time	–1.84	–7.22	<.001
time^2^	1.69	6.64	<.001

^a^Standardized coefficients.

In Figure 1, the slope change appears to have happened at the end of 2006 and beginning of 2007, circled in red. To examine the trend before and after the apparent slope change, Prais-Winsten regressions were conducted for the 2 periods: 2004-2006 (36 months) and 2007-2020 (162 months). In addition, due to improvements in Google’s algorithms for search volume from 2011, the trend from 2011 to 2020 was also examined. The analyses are presented in Table 2.

**Table 2:** Prais-Winsten regression examining the effect of time on monthly search volume during 3 different periods of analysis.

Predictor: time	β^a^	*t*	*P* value
2004 to 2006	–0.53	–3.57 (*df*=33)	.001
2007 to 2020	0.51	7.43 (*df*=159)	<.001
2011 to 2020	0.45	5.27 (*df*=111)	<.001

^a^Standardized coefficients.

From 2004 to 2006, the Durbin Watson statistic was 1.91. The autocorrelation coefficient was –0.17 (SE 0.17). For the overall model, *R*^2^ was 0.28 (SE 16.08). Time demonstrated a negative linear effect on search volume (β=–0.53, *t*_33_=–3.57, *P*=.001), suggesting a reduction in monthly search volume during this period.

From 2007 to 2020, the Durbin Watson statistic was 1.91. The autocorrelation coefficient was –0.03 (SE 0.08). For the overall model, *R*^2^ was 0.26 (SE 3.25). Time demonstrated a positive linear effect on search volume (β=0.51, *t*_159_=7.43, *P*<.001), suggesting that monthly search volume exhibited a positive linear trend from 2007 to 2020.

From 2011 to 2020, the Durbin Watson statistic was 1.99. The autocorrelation coefficient was –0.07 (SE 0.10). For the overall model, *R*^2^ was 0.20 (SE 3.08). Time demonstrated a positive linear effect on search volume (β=0.45, *t*_111_=5.27, *P*<.001), suggesting a positive linear trend consistent with that from 2007 to 2020, despite improvements in Google Trend’s algorithms.

### Cross-Correlation Between Annual Search Volume and Disease Burden Indicators (2004-2017)

Table 3 presents cross-correlations between annual COPD search volume and disease burden indicators at lags 0, –1, and 1. There were positive correlations between search volume and prevalence, incidence, and YLD at lag 0, suggesting that these pairs of variables were contemporaneously correlated. There were also positive correlations between search volume and prevalence, as well as between search volume and YLD at lag 1, suggesting that higher prevalence and YLD coincided with higher search volume 1 year later. Correlations at all other lags were nonsignificant.

**Table 3:** Cross-correlation analysis of annual chronic obstructive pulmonary disease (COPD) search volume and COPD disease burden indicators (2004-2017).

Disease burden indicator	Annual search volume		
	Lag –1	Lag 0	Lag 1
Prevalence	0.31	0.84^a^	0.60^a^
Incidence	0.36	0.92^a^	0.50
Disability-adjusted life years (DALY)	0.04	0.06	0.49
Years living with disability (YLD)	0.29	0.84^a^	0.59^a^
Years of life lost (YLL)	–0.00	0.08	0.41
Mortality	–0.01	0.00	0.40

^a^Exceeds the 95% CI threshold.

## Discussion

This study examined the trends in OHISB for COPD in Singapore using Google search volume data between 2004 and 2020 and compared them with the trends in disease burden indicators for the same time period.

### Principal Findings

The first objective was to examine the trend in search volume in COPD from 2004 to 2020. During this period, the trend in COPD search volume was curvilinear, with a slope change at the end of 2006. Further analyses examining the trends before and after this slope change revealed a downward trend from 2004 to 2006 and an upward trend from 2007 to 2020. The downward trend from 2004 to 2006 was surprising. We speculate the reason to be noisy data due to low and inconsistent search volume from 2004 to 2006, a period during which personal computing and high-speed internet connectivity were growing rapidly but not yet widespread in Singapore. From 2007 to 2020, the search volume exhibited a positive linear trend. A search of news and events from 2004 to 2020 revealed no significant events (eg, COPD health campaigns) to explain this trend. Our finding contrasts with an analysis of global data that showed no change in the trend in COPD OHIBS from 2004 to 2018 [15] and highlights the value of adopting a country-specific approach for revealing patterns that might be diluted when countries are aggregated.

The second research objective was to compare the trend in Google search volume with the disease burden indicators of COPD. From 2004 to 2017, there were positive correlations between search volume and several COPD disease burden indicators (prevalence, incidence, YLD) in the GBD study, suggesting that the trend in COPD search volume reflected the increase in some COPD morbidity indicators in the country. Search volume was not correlated with COPD mortality and YLL due to the disease, suggesting that Google search volume might be driven by the need to manage the illness for those living with COPD. This is consistent with the observation that improved health care has prolonged the living years of patients living with chronic illnesses, such as COPD.

The positive correlations between OHISB trends and COPD disease burden indicators (particularly prevalence and YLD) suggest that increased prevalence of COPD morbidity may have manifested in increased OHISB. COPD patients might be searching for health information on the internet to cope with their illness. Some of these increased searches might also have been performed by carers and family members of patients with COPD [20] or health care workers, especially those still in training. Patients with COPD appear to rely on their physicians as their primary source of information [12], but OHISB may still have a significant role when access to formal health care is limited. In Singapore, it is common for consultations with general practitioners to last for ≤5 minutes, so patients need to be well informed to optimize the brief consultation. Several patient-related barriers to timely COPD diagnoses have been reported in the literature. Patients tend to adapt to and underreport their respiratory symptoms, leading to potential underdiagnosis of COPD [21]. Older patients may also mistake symptoms such as shortness of breath as normal signs of aging [22]. Increased awareness of the significance of these symptoms among high-risk patients (eg, smokers) can increase the likelihood of a more timely diagnosis and prompt management of exacerbations.

Recommendations should be given to providers of online health information to make their materials more credible and user-friendly, reducing the barrier to timely health care seeking. Health knowledge can empower COPD patients to actively manage their own illnesses and make informed decisions about their conditions. For patients who seek health information online regularly, health care professionals can recommend trustworthy websites to complement their illness management [12].

In adopting a country-specific approach, this study seeks to present a nuanced picture of OHISB in Singapore’s rapidly aging population with high internet penetration and increasing COPD burden. Heterogeneous patterns may be concealed when countries are aggregated for analysis. Furthermore, findings from single-country studies are more useful for formulating policies, which need to be tailored to the specific conditions of the target country.

### Limitations

The findings of this study should be considered in the light of its limitations. First, while Google is the most popular search engine by a large margin, it nevertheless does not encompass all OHISB for COPD. Future research needs to study COPD OHISB on other platforms, such as social media, to understand how those OHISB change in accordance with COPD morbidity. Second, although Google indicated that Google Trends data should be understood as a metric of interest relative to searches on other topics, the specific way in which Google Trends data are derived is still unknown. Third, we note that the positive linear trend in COPD search volume is only moderate, and the interest level in this condition is low relative to some other health conditions. Finally, we acknowledge that this study is observational in nature, and the observed relationships were associative rather than causal. An additional analysis of search volume data on tuberculosis, a condition with a mostly static disease burden in Singapore, also revealed an upward trend, but to a lesser degree than COPD, suggesting a possible general increase in OHISB across medical conditions. Hence, increasing disease burden may be only one of the many factors driving OHISB in COPD.

### Conclusion

Using Google search data, this study found an increasing trend in OHISB for COPD from 2007 to 2020 in Singapore, consistent with the increases in COPD morbidity estimates over the same time period. This suggests increasing engagement in OHISB for COPD in the population, many of whom may be COPD patients and their carers. The COPD disease burden is increasing, and timely seeking of health care is imperative for its prevention, early detection, and management. Greater public awareness is essential for minimizing the disease burden. Therefore, improving access to high-quality, web-based information on COPD is recommended for fulfilling COPD patients’ information needs and improving their health outcome.

## Abbreviations

COPD: chronic obstructive pulmonary disease
DALY: disability-adjusted life years
GBD: Global Burden of Disease Study
ICC: intraclass correlation coefficient
OHISB: online health information–seeking behavior
RSI: relative search interest
TARIPH: Academic Respiratory Initiative for Pulmonary Health
YLD: years living with disability
YLL: years of life lost
